# The Prevalence of Dry Eye Disease and Related Factors Among Adult Patients Attending Primary Healthcare Centers in Riyadh, Saudi Arabia

**DOI:** 10.7759/cureus.31400

**Published:** 2022-11-12

**Authors:** Mohammed M AlMarshedi, Sulaiman A Alshammari

**Affiliations:** 1 Family Medicine, Second Cluster, Ministry of Health, Riyadh, SAU; 2 Family and Community Medicine, College of Medicine, King Saud University, Riyadh, SAU

**Keywords:** saudi arabia, riyadh, mcmonnies questionnaire, dry eye syndrome, dry eye disease

## Abstract

Objective

In this study, we aimed to determine the prevalence of dry eye disease (DED) and its related factors among adult patients in Riyadh, Saudi Arabia.

Methodology

We conducted a cross-sectional study based on a pre-designed, validated questionnaire (the McMonnies questionnaire) at primary healthcare centers in Riyadh in January-May 2019. Participants were classified into two groups: those with and without a diagnosis of DED. Factors related to the diagnosis were derived using univariate analysis. A backward stepwise logistic regression model was used to further explore predictors of DED and included all variables that were significant in the univariate analysis.

Results

A total of 276 patients participated in this study. About half (53.3%) were males, and 47.8% were aged 25-45 years. While more than two-thirds of the participants (70.3%) had some symptoms indicative of DED, only 42% were diagnosed with DED. A higher prevalence of DED was found in females. About half (47.5%) had been prescribed eye drops or other treatments for dry eyes. Univariate analysis showed a significant correlation between the incidence of DED and living in southern Riyadh (p=0.017). Additionally, all age groups were significantly associated with DED, and a higher prevalence was reported in those older than 45 years. Backward stepwise logistic regression revealed that using dry eye drops was among the most significant predictors of DED [odds ratio (OR): 339, 95% confidence interval (CI): 73.2-1577.3, p<0.001].

Conclusion

Based on our findings, DED was more common among women and with advancing age. DED was associated with the presence of dry eye symptoms, arthritis, usage of dry eye drops, and living in southern Riyadh. There is a need to design and implement health awareness programs to prevent such medical issues among the population.

## Introduction

Dry eye disease (DED), or keratoconjunctivitis sicca, is a growing public health problem worldwide. DED is one of the most frequent causes of ophthalmological morbidities. It is a multifactorial disease involving tears and the ocular surface. The symptoms of DED usually include discomfort due to blurred vision, tear film instability, burning, eye redness, dryness, and grittiness or soreness that worsens throughout the day [1]. The manifestations of DED range from subtle but constant eye irritation to significant inflammation and scarring of the eye's front surface [2]. DED is a chronic ocular surface disease widely encountered in ophthalmic practice. It has significant implications for public health, including increased healthcare costs and a negative impact on the quality of life due to its effect on vision. Visual disruption may interfere with daily activities, such as reading, television watching, computer work, and driving. The cost of managing DED in healthcare organizations in the United States is estimated to be US $700,000 per million patients [3]. Furthermore, the total annual healthcare cost has been reported to range from US $270,000 in France to US $1.10 million in the United Kingdom for every 1,000 DED patients managed by ophthalmologists [4]. Thus, determining the magnitude of the problem may help decision-makers and healthcare professionals design an eye-health promotion program that could prevent the occurrence of DED among the general public.

DED can occur due to various causes, including hormonal changes, autoimmune diseases, contact lens wear, refractive surgery, certain medications, and dry or windy climates [1]. DED can occur when the complex tear production process is disrupted (i.e., decreased tear production or excessive tear evaporation) [5]. Several studies have been conducted in various parts of the world to evaluate the prevalence of DED. The prevalence of DED, as documented in large epidemiological studies, ranges from 4% to over 30% [6,7]. According to the Women's Health Study, the condition's prevalence was 7.8%, which tended to increase with age, as per interview-based screening of 36,995 subjects older than 49 years [8]. The prevalence of DED reported in the Blue Mountains study was 15.3% [9]. The Beaver Dam Study [10] and the Sipahi Eye Study [11] reported a prevalence of 14.5% and 33.7%, respectively. Tan et al. [12] reported a prevalence of 12.3% and stated that it was associated with contact lens wear, previous treatment for dry eye, unusual sensitivity of the eyes, mucous membrane dryness, and waking eye irritation. Moreover, a large study conducted in Iran reported a prevalence of 8.7% among adults and a higher prevalence among women [13]. The prevalence of DED in the hospital-based population was 19.2% and was associated with older age and illiteracy [14]. AlShamrani et al. [15] reported a prevalence of 32.1% and stated that the condition was associated with female sex, age >55 years, cigarette smoking, and the presence of diabetes mellitus. In contrast, Bukhari et al. found no correlation between DED and age or sex, with a prevalence of 93.2% in their study [16]. There is a paucity of literature on DED in Saudi Arabia. Although our hypothesis was that DED was common in Riyadh, we did not have a clear picture of its prevalence and risk factors. In light of this, the present study aimed to evaluate the prevalence of DED and related factors among adults in Riyadh. Hopefully, these findings will help decision-makers and health professionals design intervention programs to facilitate the early detection and management of DED.

## Materials and methods

This cross-sectional study was based on a predesigned and validated questionnaire (McMonnies questionnaire). The questionnaire scale ranged from 0 to 45, with a score of 14 or higher suggesting the presence of DED [17]. The investigators conducted this study at Riyadh's primary healthcare centers between January and May 2019. They employed a multistage sampling technique to include patients of both sexes aged >18 years. Patients with active eye infections, evidence of ocular chemical or thermal burns, those who had ocular surgery within six months prior to screening, and pregnant or lactating women were excluded from the study. Informed consent was obtained from all the participants. All the collected data were treated with strict confidentiality.

The DEQ is a validated and established questionnaire with high sensitivity and specificity and is widely used to screen for DED [17,18]. This questionnaire was originally developed in English and has been translated into several foreign languages, but an Arabic version was not available. Therefore, we translated this questionnaire into Arabic according to the guidelines set by Beaton et al. [19].

The study was approved by the Institutional Review Board of Health Sciences Colleges Research on Human Subjects, King Saud University College of Medicine (IRB approval of Research Project No. E-18-3272). A pilot study was conducted with 30 participants visiting a nearby primary healthcare center to evaluate the clarity and reliability of the questionnaire.

Data were analyzed using SPSS Statistics version 22 (IBM Corp., Armonk, NY). Continuous variables were expressed as mean ± standard deviation, and categorical variables were expressed as percentages. The chi-square test and Fischer's exact test were used for categorical variables. Backward stepwise logistic regression analysis was used to explore the predictors of DED. Differences were considered statistically significant at p<0.05.

## Results

A total of 276 patients participated in this study. Approximately equal proportions hailed from the city's western, southern, eastern, central, and northern areas (19.2%, 20.7%, 21%, 19.2%, and 19.9%, respectively). More than half (53.3%) were males. Most (47.8%) were in the age group of 25-45 years (Table 1).

**Table 1:** Demographics of the study population (n=276)

Variables	Number (%)
Patient residence (regions of Riyadh)	
West	53 (19.2)
South	57 (20.7)
East	58 (21)
Central	53 (19.2)
North	55 (19.9)
Age (years)	
<25	70 (25.4)
25–45	132 (47.8)
>45	74 (26.8)
Sex	
Male	147 (53.3)
Female	129 (46.7)

While approximately half (47.5%) had been prescribed eye drops or other treatments for DED, 38.8% had not, and 13.8% were uncertain. More than two-thirds of the participants (70.3%) had some symptoms that might be indicative of DED. These symptoms included soreness, scratchiness, dryness, grittiness, and burning. Regarding the frequency of symptom occurrence, about half of the participants reported that they experienced these symptoms intermittently, while 27.5% never had symptoms, and 9.8% constantly had these symptoms. Only 29% of participants were not sensitive to cigarette smoke, smog, air conditioning, or heating, while more than one-third reported that their eyes were sensitive all the time or some of the time to these factors. In addition, the eyes of a quarter of the participants (25%) became red and irritated when swimming in chlorinated fresh water, and those of a further 26.8% were sometimes irritated. The most frequent medications used were antihistamine eye drops and diuretics. Other medications included sleeping tablets, tranquilizers, and medicines for digestive problems and high blood pressure. Approximately a quarter of the participants had arthritis. While 37.3% had never experienced dryness of the nose, mouth, throat, chest, or vagina, most of the participants had sometimes experienced these symptoms. Only approximately one-fifth of participants had thyroid abnormalities. Similar proportions reported that they slept with their eyes partly open and had eye irritation when waking from sleep. Finally, 42% of the participants were diagnosed with DED (Table 2).

**Table 2:** Responses to questions in the McMonnies dry eye questionnaire (n=276) *McMonnies score

Questions	Number (%)
Q1. Have you ever had drops prescribed or other treatments for dry eyes?	
Yes	131 (47.5)
No	107 (38.8)
Uncertain	38 (13.8)
Q2. Do you experience any of the following?	194 (70.3)
Soreness	14 (5.1)
Scratchiness	73 (26.4)
Dryness	81 (29.3)
Grittiness	15 (5.4)
Burning	43 (15.6)
Q3. How often do your eyes have these symptoms?	
Never	76 (27.5)
Sometimes	135 (48.9)
Often	38 (13.8)
Constantly	27 (9.8)
Q4. Do you regard your eyes as being especially sensitive to cigarette smoke, smog, air conditioning, or heating?	
Yes	97 (35.1)
No	80 (29)
Sometimes	99 (35.9)
Q5. Do your eyes easily become red and irritated when swimming in chlorinated fresh water?	
Not applicable	55 (19.9)
Yes	69 (25)
No	78 (28.3)
Sometimes	74 (26.8)
Q6. Are your eyes dry and irritated the day after drinking alcohol?	
Not applicable	184 (66.7)
Yes	15 (5.4)
No	75 (27.2)
Sometimes	2 (0.7)
Q7. Medications used?	
Antihistamine eye drops	58 (21)
Diuretics	54 (19.6)
Sleeping tablets	18 (6.5)
Tranquilizers	9 (3.3)
Oral contraceptives	0 (0)
Medication for digestive problems	19 (6.9)
Medication for high blood pressure	10 (3.6)
Others	21 (7.6)
Q8. Do you have arthritis?	
Yes	72 (26.1)
No	186 (67.4)
Uncertain	18 (6.5)
Q9. Do you experience dryness of the nose, mouth, throat, chest, or vagina?	
Never	103 (37.3)
Sometimes	116 (42)
Often	49 (17.8)
Constantly	8 (2.9)
Q10. Do you suffer from thyroid abnormality?	
Yes	55 (19.9)
No	158 (57.2)
Uncertain	63 (22.8)
Q11. Are you known to sleep with your eyes partly open?	
Yes	62 (22.5)
No	192 (69.6)
Uncertain	22 (8)
Q12. Do you have eye irritation when you wake up from sleeping?	
Yes	61 (22.1)
No	193 (69.9)
Uncertain	22 (8)
Diagnosis of dry eye	
Yes (>14)*	116 (42)
No (≤14)*	160 (58)

The participants were classified into two groups: the first group included participants diagnosed with DED (42%) and the second group included those not diagnosed with DED (Table 3). Univariate analysis demonstrated a significant correlation between the incidence of DED and living in southern Riyadh (p=0.017). There was also a significant association between DED status and different age groups (<25, 25-45, and >45 years) (p=0.003, 0.005, and <0.001, respectively): most of the DED patients were more than 45 years old. The prevalence of DED was higher among females and those who had been prescribed eye drops or other treatments for DED. It was also higher among those with symptoms (p<0.001), especially dryness and burning (p<0.001 and 0.003, respectively). The absence and the occasional occurrence of sensitivity to cigarette smoke, smog, air conditioning, or heating (p=0.010 and 0.004, respectively) and the occurrence and absence of eye irritation when swimming in chlorinated fresh water (p=0.005 and <0.001, respectively) were also associated with DED status. The use of antihistamine eye drops and tranquilizers (p=0.023 and 0.009, respectively), the presence of arthritis (p<0.001), dryness of the nose, mouth, throat, chest, or vagina (p<0.001,<0.001, and 0.001, respectively), the presence and absence of thyroid abnormalities (p<0.001), and eye irritation when waking from sleep were also associated with DED.

**Table 3:** Univariate analysis to explore determinants (risk factors) of dry eye *McMonnies score. **Significant p-value

Variables	Diagnosis of dry eye		P-value
	Yes (>14)*	No (≤14)*	
	(n=116)	(n=160)	
Region of residence			
West	21 (18.1)	32 (20)	0.693
South	16 (13.8)	41 (25.6)	0.017^**^
East	24 (20.7)	34 (21.3)	0.91
Central	26 (22.4)	27 (16.9)	0.249
North	29 (25)	26 (16.3)	0.072
Age (years)			
<25	19 (16.4)	51 (31.9)	0.003^**^
25–45	44 (37.9)	88 (55)	0.005^**^
>45	53 (45.7)	21 (13.1)	<0.001^**^
Sex			
Male	41 (35.3)	106 (66.3)	
Female	75 (64.7)	54 (33.8)	<0.001^**^
Q1. Have you ever had drops prescribed or other treatments for dry eyes?			
Yes	107 (92.2)	24 (15)	<0.001^**^
No	0 (0)	107 (66.9)	<0.001^**^
Uncertain	9 (7.8)	29 (18.1)	0.014^**^
Q2. Presence of any symptoms	116 (100)	78 (48.8)	<0.001^**^
Soreness	9 (7.8)	5 (3.1)	0.083
Scratchiness	37 (31.9)	36 (22.5)	0.081
Dryness	55 (47.4)	26 (16.3)	<0.001^**^
Grittiness	8 (6.9)	7 (4.4)	0.362
Burning	27 (23.3)	16 (10)	0.003^**^
Q3. How often do your eyes have these symptoms?			
Never	0 (0)	76 (47.5)	<0.001^**^
Sometimes	64 (55.2)	71 (44.4)	0.077
Often	25 (21.6)	13 (8.1)	0.001^**^
Constantly	27 (23.3)	0 (0)	<0.001^**^
Q4. Do you regard your eyes as being especially sensitive to cigarette smoke, smog, air conditioning, or heating?			
Yes	39 (33.6)	58 (36.3)	0.652
No	24 (20.7)	56 (35)	0.010^**^
Sometimes	53 (45.7)	46 (28.7)	0.004^**^
Q5. Do your eyes easily become red and irritated when swimming in chlorinated fresh water?			
Not applicable	33 (28.4)	22 (13.8)	0.003^**^
Yes	39 (33.6)	30 (18.8)	0.005^**^
No	13 (11.2)	65 (40.6)	<0.001^**^
Sometimes	31 (26.7)	43 (26.9)	0.978
Q6. Are your eyes dry and irritated the day after drinking alcohol?			
Not applicable	70 (60.3)	114 (71.3)	0.058
Yes	7 (6)	8 (5)	0.708
No	37 (31.9)	38 (23.8)	0.133
Sometimes	2 (1.7)	0 (0)	0.096
Q7. Medications used			
Antihistamine eye drops	32 (27.6)	26 (16.3)	0.023^**^
Diuretics	29 (25)	25 (15.6)	0.053
Sleeping tablets	7 (6)	11 (6.9)	0.78
Tranquilizers	0 (0)	9 (5.6)	0.009^**^
Oral contraceptives	0 (0)	0 (0)	….
Medication for duodenal ulcer	0 (0)	0 (0)	…..
Medication for digestive problems	8 (6.9)	11 (6.9)	0.994
Medication for high blood pressure	3 (2.6)	7 (4.4)	0.432
Others	6 (5.2)	15 (9.4)	0.194
Q8. Do you have arthritis?			
Yes	50 (43.1)	22 (13.8)	<0.001^**^
No	56 (48.3)	130 (81.3)	<0.001^**^
Uncertain	10 (8.6)	8 (5)	0.229
Q9. Do you experience dryness of the nose, mouth, throat, chest, or vagina?			
Never	25 (21.6)	78 (48.8)	<0.001^**^
Sometimes	44 (37.9)	72 (45)	0.24
Often	39 (33.6)	10 (6.3)	<0.001^**^
Constantly	8 (6.9)	0 (0)	0.001^**^
Q10. Do you suffer from thyroid abnormality?			
Yes	47 (40.5)	8 (5)	<0.001^**^
No	46 (39.7)	112 (70)	<0.001^**^
Uncertain	23 (19.8)	40 (25)	0.312
Q11. Are you known to sleep with your eyes partly open?			
Yes	46 (39.7)	16 (10)	<0.001^**^
No	62 (53.4)	130 (81.3)	<0.001^**^
Uncertain	8 (6.9)	14 (8.8)	0.575
Q12. Do you have eye irritation when you wake up from sleeping?			
Yes	59 (50.9)	2 (1.3)	<0.001^**^
No	42 (36.2)	151 (94.4)	<0.001^**^
Uncertain	15 (12.9)	7 (4.4)	0.010^**^

Backward stepwise logistic regression analysis was used to explore the predictors of DED (Table 4). All the significant variables in the univariate analysis were included in the model. This model revealed that the only significant predictors of DED were the use of eye drops [Q1, odds ratio (OR): 339, 95% confidence interval (CI): 73.2-1577.3, p<0.001], the presence of arthritis (Q2, OR: 60.5, 95% CI: 12.2-299.3, p<0.001), and dryness of the nose, mouth, throat, chest, or vagina (Q3, OR: 13.0, 95% CI: 4.9-34.4, p<0.001).

**Table 4:** Multivariate analysis to explore predictors (risk factors) of dry eye β: beta coefficient; OR: odds ratio; CI: confidence interval

	β	OR	95% CI	P-value
Q1. Use of eye drops	5.8	339.9	73.2–1577.3	<0.001
Q8. Presence of arthritis	4.1	60.5	12.2–299.3	<0.001
Q9. Dryness of nose, mouth, throat, chest, or vagina	2.6	13.0	4.9–34.4	<0.001
Constant	-6.5	0.002		

## Discussion

DED is among the most common conditions encountered by ophthalmologists and is one of the primary reasons behind patients seeking care. Unfortunately, neither the prevalence of DED in Riyadh nor the factors contributing to its development have been identified. Addressing these factors was the primary focus of our investigation.

Our findings showed that DED affected 42% of the total participants. This finding is higher than that reported in other studies [6,11,20]. The prevalence of DED varies considerably between epidemiological studies. It can be as low as 7.4% or as high as 33.7%, depending on how the disease is defined and diagnosed and which population is surveyed [6,11,20].

The high prevalence of DED found in this study could be attributed to several factors, including the hot and dusty climate of Riyadh. Riyadh is sweltering and arid in summer, with temperatures averaging 39 °C from May 14 to September 24. July in Riyadh experiences average temperatures of 43 °C and 30 °C, typical of a hot desert climate, while winters are cool and dry. The coldest season lasts from November 27 to February 26, with an average temperature of 25 °C or less. January is Riyadh's coldest month, with the temperature averaging between 10 °C and 20 °C. The average annual temperature is from 9 °C to 43°C, seldom below 5 °C or over 45 °C. Summer dust storms can restrict vision up to 10 meters. Riyadh's annual rainfall ranges from 7 millimeters in March to 0 millimeters in July. The yearly average humidity is 29%. It ranges from 50.0% in January to 14.0% in August. In addition, the high morbidity caused by chronic diseases such as diabetes and hypertension may have played a significant role in causing DED among the residents. There was a statistically significant correlation between the presence of DED and age; most patients with DED were over 45 years old. This finding is in line with the results of other studies that demonstrated an increase in the prevalence of DED with increasing age [17,21,22]. However, no significant association between age and the development of DED was found [6,16,23,24].

There is also a lack of consensus among studies concerning the association between sex and DED. We discovered that DED was more prevalent among women than men (p=0.001). This finding is consistent with that of a study on an elderly Chinese population in Taiwan [6] and the Beaver Dam Eye Study conducted in Wisconsin, United States [22]. In contrast to the findings of a study conducted in Indonesia, which found that the prevalence of DED is 1.4 times higher in men than in women [11], various other studies, such as the one conducted in Jeddah, Saudi Arabia [16], the one among the Hispanic population in the US [21], one performed in Brisbane, Australia [17], and a study from Bangkok, Thailand [23], discovered no significant correlation between the development of DED and sex. These differences among studies in terms of the association of DED development with advancing age and the fact that more cases of DED were found in women in our study than in others in the literature may be attributed to environmental, biological, or ethnic factors.

Among our subjects, 33.6% were affected by cigarette smoke, smog, air conditioning, heating, and swimming in freshwater that had been treated with chlorine. All of them had DED, even though there was no clear correlation between the two. The tear film can become disrupted due to irritation caused by environmental factors such as smoke. Previous research has found that smoking was the second most common behavioral factor associated with DED, with a statistically significant association with DED as a risk factor [16,25]. Another study found that 17.6% of the DED participants were smokers [22]. In addition, research conducted by other groups has discovered a connection between DED and smoking rates, with an average of 34.1% among their respective populations [11].

In addition, DED was associated with the presence of symptoms of dry eye, treatment for dry eye, living in the southern part of Riyadh, and the presence of arthritis. The logistic regression model results showed that the only significant predictors of DED were eye drop use, arthritis, and dryness of the nose, mouth, throat, chest, and vagina. The association between arthritis and DED was consistent with previous findings [26]. It is possible to link the association of DED with rheumatoid arthritis to autoimmune processes in the body; however, this does not apply to osteoarthritis. One explanation could be that people diagnosed with osteoarthritis have lower pain thresholds than others [27]. In patients who reported symptoms of DED, we observed an increase in sensitivity to thermal pain. Subjects may begin to complain about their degenerative joint disease (DED) and osteoarthritis earlier than others who have similar abnormalities because of their increased pain sensitivity. This correlation suggests that DED and pain have a common underlying cause. Another interesting observation was the association of vaginal dryness with DED. This may be attributed to the increasing age and hormone imbalance. Another study found a rising prevalence of DED among postmenopausal women [28].

Antihistamine eye drops and diuretics were the most frequently prescribed medications in our cohort. Other medications included sleeping pills, tranquilizers, medication for digestive issues, and those for both high blood pressure and high cholesterol. Based on previous research findings, certain medications, including antihistamines, diuretics, and tranquilizers, may have played a role in some of the associations we found [29]. Recent research has found that a moderate intake of vitamin C (antioxidant) might serve as a preventative and therapeutic strategy for dry eyes and inflammatory symptoms. It also has an excellent safety profile [30].

We recommend further studies involving a larger and more representative sample size based on a combination of self-reported symptoms and clinical evaluation from multiple regions in Saudi Arabia, which would enable researchers to investigate patients' quality of life and better understand the various factors associated with DED in Saudi Arabia. Furthermore, health authorities and professionals could use these findings to design and implement health awareness programs to prevent such medical issues in the general population.

Limitations

This study has a few limitations. Primarily, we relied on a valid screening tool for participants to self-report their symptoms of DED rather than a clinical evaluation of signs or a combination of the two. Although valid self-report questionnaires are vital tools for determining the prevalence of DED in population-based studies, there is a possibility that this could introduce recall bias. Additionally, because the study was conducted in Riyadh, the results may not apply to other areas of Saudi Arabia, particularly rural areas.

## Conclusions

Based on our findings, DED was common in our cohort and affected 42% of our participants representing Riyadh's adult population. It was more prevalent in women and associated with increasing age, particularly after 45 years. In addition, more than two-thirds of participants with DED reported soreness, scratchiness, dryness, grittiness, and burning. The same proportion of participants was sensitive to cigarette smoke, smog, air conditioning, heating, and swimming in chlorinated freshwater. Furthermore, DED was associated with dry eye symptoms, dry eye treatment, living in the southern part of Riyadh, and arthritis. The logistic regression model revealed that the use of eye drops, the presence of arthritis, and dryness of the nose, mouth, throat, chest, or vagina were the only significant predictors of DED. The most frequent medications used were antihistamine eye drops and diuretics. Other medications included sleeping tablets, tranquilizers, and those for digestive problems and high blood pressure. Further studies with larger sample sizes based on a combination of self-reported symptoms and clinical evaluations from multiple regions will provide a better understanding of the various factors related to DED in Saudi Arabia and allow researchers to investigate the patients' quality of life. Furthermore, health authorities and professionals could use these findings to design and implement health awareness programs to help prevent the occurrence of such medical conditions among the population.
